# Reducing the risk of invasive forest pests and pathogens: Combining legislation, targeted management and public awareness

**DOI:** 10.1007/s13280-015-0748-3

**Published:** 2016-01-07

**Authors:** Maartje J. Klapwijk, Anna J. M. Hopkins, Louise Eriksson, Maria Pettersson, Martin Schroeder, Åke Lindelöw, Jonas Rönnberg, E. Carina H. Keskitalo, Marc Kenis

**Affiliations:** Department of Ecology, Swedish University of Agricultural Sciences, Box 7044, 750 07 Uppsala, Sweden; Centre of Excellence for Climate Change, Woodlands and Forest Health, Murdoch University, WA 6150 Perth, Australia; Department of Forest Mycology and Plant Pathology, Swedish University of Agricultural Sciences, Uppsala, Sweden; Department of Geography and Economic History, Umeå University, 901 87 Umeå, Sweden; Department of Business Administration, Technology and Social Sciences, Luleå University of Technology, 971 87 Luleå, Sweden; Department of Ecology, SLU, Box 7044, 750 07 Uppsala, Sweden; Southern Swedish Forest Research Centre, Swedish University of Agricultural Sciences, 230 53 Alnarp, Sweden; CABI Europe-Switzerland, 1 Rue des Grillons, 2800 Delémont, Switzerland

**Keywords:** Biosecurity, European Union, Pathways, Plant health, Plants for planting, World trade organisation

## Abstract

Intensifying global trade will result in increased numbers of plant pest and pathogen species inadvertently being transported along with cargo. This paper examines current mechanisms for prevention and management of potential introductions of forest insect pests and pathogens in the European Union (EU). Current European legislation has not been found sufficient in preventing invasion, establishment and spread of pest and pathogen species within the EU. Costs associated with future invasions are difficult to estimate but past invasions have led to negative economic impacts in the invaded country. The challenge is combining free trade and free movement of products (within the EU) with protection against invasive pests and pathogens. Public awareness may mobilise the public for prevention and detection of potential invasions and, simultaneously, increase support for eradication and control measures. We recommend focus on commodities in addition to pathways, an approach within the EU using a centralised response unit and, critically, to engage the general public in the battle against establishment and spread of these harmful pests and pathogens.

## Introduction

Pest and pathogen invasions are closely linked to global trade in plants for planting and wood products; this trade has greatly intensified in recent decades (Shirley and Kark 2006; Westphal et al. 2008). Historically, Europe has been less affected by pest and pathogen invasions than, for example, North America and Australasia (Niemelä and Mattson 1996). However, globalisation and changes in trade relations have led to increasing accidental introductions of invasive species in Europe (Santini et al. 2013). The number of alien species establishing annually in Europe has increased twofold between 1950 and 2009 for invertebrate species (Roques et al. 2009) and fourfold between 1900 and 2009 for fungal species (Desprez-Loustau 2009). Invasive species are often divided into two categories: (1) species that pose threats to ecosystems by altering species composition, and (2) species that pose a threat to human interest, mostly economically. The latter are referred to as invasive pests and pathogens (IPPs) and are the focus of this paper. Protecting forests from risks posed by these IPPs is essential. That over 100 scientists have signed the Montesclaros Declaration, which calls “to phase out all trade in plants and plant products determined to be of high risk to forested ecosystems but of low overall benefit”^1^ recognising the ineffectiveness of the current phytosanitary practises, puts even more emphasis on the urgency of the matter.

This study reviews potential means to address IPPs in European forests. We focus on IPPs affecting forestry and wood trade, wood products and plants in the European Union (EU); legislative complexities regarding prevention, interception and control within the EU; possibilities to centralise management responsibility and the role of public awareness for control programmes.

## Background

IPPs are responsible for losses of trees and/or production in both urban areas and commercial forests (Moore 2005). Damage by invasive tree borers in the USA costs ca. US$2000 million annually (Aukema et al. 2011). In Europe, total annual costs of invasive species have been roughly estimated at nearly €10 000 million (Kettunen et al. 2009) but few corresponding data are available for forest IPPs (Kenis and Branco 2010). Damage by the invasive fungi *Ophiostoma ulmi* and *O. novo*-*ulmi* (causal agents of Dutch Elm Disease) in Sweden has reportedly cost €9–228 million annually since 1979 (Gren et al. 2009). This is a small percentage of the forest sector’s €109 000 million annual production value (Forests Europe 2011), but purely financial analyses of the effect of IPPs neglect potentially greater costs of damage to ecosystem services, amenities and other ecological values (Kenis et al. 2009; Lambertini et al. 2011). Thus, protection from invasion risks is important both from ecological and economical perspective (Parker et al. 1999; Aukema et al. 2011). Other examples of devastating invasions are *Phytophthora ramorum*, the pathogenic agent responsible for sudden oak and larch death (Brasier and Webber 2010; Hansen 2015), Asian and Citrus Longhorned beetle (resp. *Anoplophora glabripennis* and *A. chinensis*) reproducing in a wide range of deciduous trees causing tree mortality (Haack et al. 2010) and a well-documented case of invasion in Europe is the establishment of the Pinewood Nematode (*Bursaphelenchus xylophilus*), vector of Pine wilt disease in Europe (Box 1).

**Table:** 

**Box 1** EU responses to the pinewood nematode The pinewood nematode (PWN) is a causal agent of pine wilt disease (PWD), a serious threat to native pine forests in eastern Asia and Europe (Dwinell 1997; Togashi and Shigesada 2006). The nematodes and its vectors (Monochamus beetles; Linit 1988) develop in coniferous trees, PWN juveniles move into the respiratory system of newly developed beetles, which emerge and subsequently feed on the bark of living conifers. PWN often enters the tree using feeding wounds made by the beetles. Female beetles then lay eggs in the bark of dying or recently cut trees, through which PWN colonises the wood. In North America, where PWN is native and seldom kills trees, this saprophytic lifecycle dominates. However, in other areas the beetles and PWN can colonise and kill numerous living trees. In 1984, the Finnish Plant Quarantine Service detected PWN in wood chips imported from North America (Rautapää 1986). Consequently, import of untreated conifer wood to Europe from PWN-infested areas was banned. However, in 1999 PWD was detected in trees in Portugal (Mota et al. 1999), carried by the native *M. galloprovincialis*, which is not present in North America or Asia. At that time, the PWN seems to have been restricted to a limited area south of Lisbon. Immediately, an eradication attempt was initiated by Portuguese authorities following recommendations (and partially funded) by the EU. Nevertheless, it has spread across the entire Portuguese mainland, and been detected in both Madeira (Fonseca et al. 2012) and four times in Spanish regions bordering Portugal (Robertson et al. 2011; Vicente et al. 2012; NPPO Spain 2014). All EU Member States are required to conduct yearly surveys for PWN (Commission Decision 2012/535/EU). If detected, a demarcated area consisting of an infested zone based on a delimitation survey and a buffer zone (at least 20 km wide) must be created. Around each PWN-infested tree, all susceptible trees should be cut, removed and disposed of within a radius of 500 m (i.e. the clear-cut zone). The buffer zone will be subjected to annual inspections and all susceptible trees of low vigour will be removed from this zone. If PWN is detected in the buffer zone, the demarcated zone will be adjusted to include the infested part of the buffer zone and a new buffer zone will be established. If the annual surveys detect PWN in the demarcated area during four or more consecutive years, and eradication proves impossible, the Member State may instead decide to contain PWN (as Portugal has done).	

All of these species will have impact on the economy of the country that they invade, either directly by reducing the revenue of the country (Soliman et al. 2012), indirectly through imposed trade restrictions (Bergseng et al. 2012) or through reduced values of real estate (Aukema et al. 2011).

Reduction or management of the threat posed by IPPs depends on the potential to embed IPP consideration in international agreements, EU legislation and national law hierarchically. Currently, the legal framework of the World Trade Organisation (WTO) prevents the EU from enacting legislation that could inhibit potential trade with other countries unless there is proven economic damage. Thus, responses to IPPs have been largely reactive rather than precautionary (Pettersson and Keskitalo 2012). Similarly, EU membership generally prohibits stringent national laws that inhibit other Member States’ economy and trade. As the dependence on the forest industry varies widely within the EU, there are conflicting interests regarding legislation promoting plant health. Decentralised responsibility for surveillance and monitoring systems has resulted in widely varying intensity and efficacy throughout the community. Recently, the EU has taken positive steps to understand the potential risks associated with IPPs. One large project regarding invasive species ‘DAISIE’ (Delivering Alien Invasive Species Inventories for Europe^2^) started in 2003 and still provides up-to-date information collected by experts. Another initiative is the COST-action programmes, in which several programmes are currently active, e.g. European Information System for Alien Species (TD1209), and a global network of nurseries as early warning system against alien tree pest (Global Warning; FP1401).

Strategies against IPPs can be divided into three categories: prevention and interception, early detection and surveillance, and reporting and management (e.g. Blackburn et al. 2011). Within the EU, these strategies need to be supported by the individual member states to be successful. Increased public involvement and public understanding of threats posed by forest IPPs may increase the willingness to legislate or take action (e.g. Hulme et al. 2009b; Simberloff et al. 2013). Thus, legislative and management strategies to reduce risk in combination with measures to increase socio-political awareness of the risks could become important ways to reduce the risk of IPPs.

## Legislation, policy and management

Within the EU, invasive alien species and IPPs are regulated under two different sections of European Commission. The invasive alien species fall under the responsibility of Environment Directorate-General (DG Environment). This directorate has recently passed new legislation that entered into force 1 January 2015. In summary, the regulation states that the EU will formulate a list of invasive alien species of ‘Union Concern’ with a risk assessment for each species. It is prohibited to bring those species into the EU or breed, grow, transport, sell them or release them into the environment. In order to handle species on this list, special permits are needed. After publishing of the list, member states have 3 years to formulate an action plan for their country, containing priority pathways and ways to prevent unintentional introduction and/or spread. It is also stated that, within 18 months after publication of the list, member states must have in place a surveillance system and measure for rapid eradication up on observation of a species from the list (Regulation (EU) 1143/2014).

On the other hand, the legislation within the EU regarding the IPPs falls under responsibility of Directorate-General Health and Food Safety (DG SANCO) that formulates the regulations regarding plant health and biosecurity. These two legislations are kept separate in their respective aim (environment, trade). Invasive alien species regulation (1143/2014, recital no 8) states that “there are over 40 Union legislative acts on animal health which include provisions in animal diseases. Moreover Council Directive 2000/29/EC includes provisions for species which are harmful to plants and plant products and Directive 2001/18/EC of (—) sets out the regime applicable to genetically modified organisms. Therefore, any new rules on invasive alien species should be aligned with and not overlap with these legislative acts—and should not apply to the organisms targeted by those legislative acts”.

The efficiency of the EU legislation regarding plant health and biosecurity has recently been evaluated in order to propose a revision for the first time in decades. The following section focuses on the legislation, policy and management related to plant health and biosecurity.

### Prevention and interception

Key pathways for IPP introductions include plants for planting, wood, wood products and wooden packaging materials (Hulme et al. 2008; Hulme 2009; Hulme and Roy 2010; Eschen et al. 2015a). To reduce risk of accidental introductions, the International Plant Protection Convention (IPPC) has formulated International Standards for Phytosanitary Measures (ISPMs).^3^

ISPM 15 (issued in 2002) states that wood packaging must be debarked and heat treated or fumigated with methyl bromide and stamped or branded with a mark of compliance prior to use. After ISPM 15 came into effect, the infestation rates in US dropped from 36–52 % to 0.11 %, which is a substantial decrease but it still means that of 13 million containers with wood packaging material more than 13 000 contain infested consignments (Haack et al. 2014). Also, the increased trade in wood chips forms a risk for accidental introductions of bark boring insects and fungal pathogens (Flø et al. 2014).

However, plants for planting have been found to be the commodity that is most likely to be infested with IPPs (Liebhold et al. 2012). Therefore, the International Plant Protection Convention has formulated ISPM 36 to specify the requirements for plants for planting and is integrating a number of previously formulated ISPM’s covering plant health, e.g. Pest Risk Analysis (ISPM 2:2007), Pest Risk Analysis for quarantine pest (ISPM 11:2004) and Pest Risk Analysis for regulated non-quarantine species (ISPM 21:2004). The aim of ISPM 36 is to create criteria for identification and application of integrated measures for the production of plants for planting for the international trade in the country of origin.

For import into the EU, sites of producers of plants for planting are subject to inspection, and both producer registration and plant passports are required (Directive 2000/29/EC). All plants imported into the EU require certification stating that they are free from harmful organisms and that phytosanitary measures stipulated by the importing country have been applied. Inspections are carried out at ports of entry but occur on a small proportion of living plants, plant material, soil and wood products that arrive in Europe (Bacon et al. 2012). The main purpose of the inspections is to verify whether shipments comply with regulations, rather than to stop potentially harmful organisms, and even then only a small proportion of the shipments can be subjected to inspection (Liebhold et al. 2012; Eschen et al. 2015a). In addition, there are large differences in inspection intensity among the EU member states (Eschen et al. 2015b). Within the EU, the shipment can be moved between countries once it is cleared for entrance at one of the inspection points. Plant and wood material from within EU member states can be moved around freely and only certain plant species need a plant passport (listed in Part A, Annex V of Directive 2000/29/EC). The variation in phytosanitary inspection of woody plants for planting increases the risk of invasion of IPPs depending on the point of entry of the EU (Eschen et al. 2015b).

Other territories have different rules. For example, in Australia and New Zealand all imported plant products have to be assessed and proved safe before permission to import the product is granted. WTO membership commits the EC (European Commission) to agreements regarding trade liberalisation. WTO members need to extensively document the threat of invasive species based on scientific evidence in order to be able to strengthen legislation around importing live plants and wood products (Pettersson and Keskitalo 2012), resulting in different levels of biosecurity for different territories, for example the EU and Australia. The General Agreement on Tariffs and Trade (GATT) and the Agreement on the Application of Sanitary and Phytosanitary Measures (the SPS agreement) are most relevant to IPPs. The central constraint of the WTO’s legal regime is the principle of national treatment (GATT Article III), stipulating that countries must treat imported and domestic goods equally.

The current core instrument in the EU legal framework for forest IPPs is Directive 2000/29/EC, which sets phytosanitary standards for trade within the EU and imports intended to prevent the introduction and spread of organisms harmful to plants or plant products. It includes a “black-list” of plants and plant products (based on recommendations by the European and Mediterranean Plant Protection Organisation, EPPO, subject to final EC decisions) that are banned from import into the EU, and procedures to apply when they are found in the EU. But these “quarantine lists” provide insufficient protection from threats posed by IPPs, as often harmful organisms that enter the EU are unknown prior to establishment (Brasier 2008). In view of the current system’s incapacity to control the increasing influx of harmful organisms as a result of globalisation of trade, the EC has submitted a proposal for a new Regulation on protective measures against pest of plants (COM (2013) 267 final), thus planning to substantially change the health regime for the first time in decades. The proposed Regulation aims to ‘prioritise, modernise, step up prevention and reinforce actions against outbreaks’, by, e.g. simplifying and harmonising plant passports, allowing for stricter measures against pests and pathogens, and addressing emerging risks from certain plants for planting from certain third countries. Thus, instead of listing harmful plant IPPs, the proposed Regulation ‘sets out the conceptual nature of quarantine pests’ and empowers the Commission to address IPPs from plants by establishing measures to control of certain pests by implementing legislative acts (COM (2013) 267 final).

The proposed Regulation is taking significant steps forward to increase measures of prevention and interception. However, precautionary assessments of high-risk commodities such as plants for planting and wood products (Richardson et al. 2010; Webber 2010) as already implemented by certain countries or regions (e.g. USDA-APHIS 2000; Biosecurity New Zealand 2006; Biosecurity Australia 2007) could play a more important role in the measures against IPPs. In addition to using risk assessments, the legislative framework should also focus on risk management by restrictions on commercial imports, such as setting maximum sizes for imported plants or banning imports of plants in soil. But this cannot be introduced without scientific evidence for their necessity. So far, progress has been made by excluding high-risk plant genera, which may help reduce import risks (Evans 2010) and import bans can be enhanced through commodity risk analyses.

Since compliance to the WTO constrains use of precautionary measures by the EC, EU regulation heavily depends on entry-, pathway- and species-based risk analyses. These analyses do not protect against non-quarantine IPPs as they are mainly focussed on known species of IPPs. Rapid responses to invading organisms are required to eradicate or contain them, once they have been observed. However, the current system often fails because it depends on investments and actions of individual Member States (Hulme 2006), neglecting the increasing costs of delays in initiating eradication measures.

For a few invasive species, e.g. the pine wood nematode (PWN, *Bursaphelenchus xylophilus*; Steiner and Buhrer 1934) currently present in Portugal, mandatory EU legislation stipulates how and when eradication attempts should be undertaken. Based on the current regulation dealing with PWN, each country needs to have a contingency plan for when the species is detected. The requirement posed by the legislation is based on a negotiation between interests of the Member States, for which the interests might be conflicting on certain occasions (Økland et al. 2010).

Hulme et al. (2009a, b) have proposed establishment of an EU agency similar to the European Centre for Disease Prevention and Control for all types of invasive species (rather than, as now, dealing with invasive alien species and IPPs separately through the Environment Directorate-General of the EC and Health and Food Safety Directorate-General, respectively). The difficulty with protection against invasions from outside the EU and containment within the EU is illustrated by the summary of responses to the PWN in Box 1. The need for a centralised agency together with improvements in detection, monitoring, reporting and management is further considered in the discussion.

### Early detection and monitoring

Early detection of IPPs—and thus active monitoring, involving regular surveys in specific areas of interest (Brockerhoff et al. 2010; Rassati et al. 2015)—is essential for both reducing eradication costs and increasing probability of success (Mehta et al. 2007). A primary difficulty is that IPPs are generally rare during early stages of incursions, but already widespread when damage is first observed. Thus, monitoring close to potential entry points (ports, airports, etc.) and in sensitive areas is critical. However, while a comprehensive surveillance system would significantly improve their capacity to respond quickly to IPP incursions, extensive resourcing and enforcement from the central authorities would be needed in order to control both entry ports as well as, for instance, plant nurseries and other potential channels through which IPPs could spread. However, the benefits of regular surveillance must be weighed against the costs (Epanchin-Niell et al. 2012), and may be minor for individual Member States, but greater for the wider EU community.

### Reporting and management

**Fig. 1:**
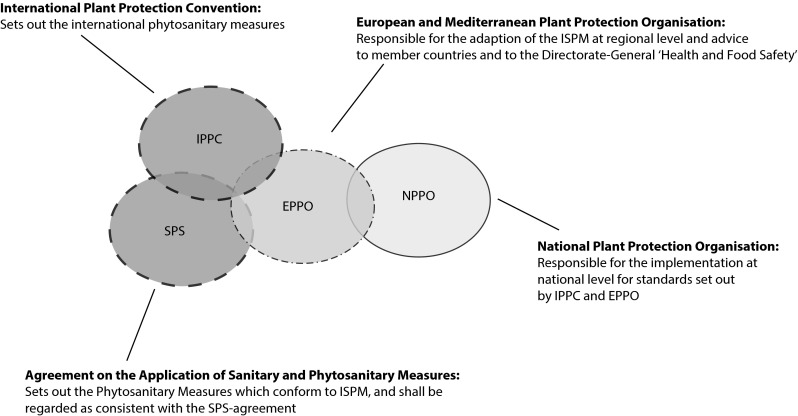
Hierarchical structure of phytosanitary organisations and their area of responsibility (figure adapted from Lopian 2005). *Dashed circles* represent global organisations, *dashed*-*dot circles* represent ‘regional’ organisations (EU +) and the *solid circle* represents national organisations. The international phytosanitary standards (ISPM) are set out by IPPC to protect plants from plant pests (insects and pathogens). The SPS agreement sets out trade-rules regarding plant health. EPPO is responsible for the adaptation of the ISPM at regional level (which is in this case EU +) and advising the member states and the European Commission. The NPPOs are responsible for implementing the standards, as formulated by EPPO, in their respective countries

Directive 2000/29/EC requires Member States to eradicate and/or prevent the spread of detected IPPs through their National Plant Protection Organisations (NPPO; for the relationship between the international IPPCs, EPPO and NPPO see Fig. 1). The EC’s Standing Committee on Plant Health (as part of the Directorate-General Health and Food Safety) is mandated to introduce measures to control spread. For new pests and pathogens, this can lead to harmonised eradication and containment measures, based on pest risk analyses, which may be co-financed by the EU. However, some countries do not consistently and promptly report detected incursions (Brasier 2008). Formal acknowledgement of harmful organisms’ presence often lags several years behind detection, allowing them to spread before eradication measures can be taken (Landeras et al. 2005; Brasier 2008). Further, as with biosecurity breaches in trade, member states that are unaware of or unwilling to report new incursions are rarely prosecuted (Brasier 2008).

Eradication can be efficient but is often costly. Since 1996, the two Asian Longhorned beetles *A. glabripennis* and *A. chinensis* have been detected many times in North America and Europe. All detections have been followed by targeted eradication programmes of various scales, depending on the evidence for establishment and size of the infestation (Haack et al. 2010). In North America, significant breeding populations of *A. glabripennis* were found in at least five US states and in Ontario. The expenditure on eradication programmes is probably over a 1000 million US$ since their start in 1997 ($396 million from 2001 to 2008 Haack et al. 2010; more than $800 million from 1997 to 2006 in the US alone; Smith and Wu 2008). As a result, *A. glabripennis* is now considered eradicated in Illinois, New Jersey and several other localities but new populations are regularly appearing, e.g. in Ontario in 2013 where the beetle had previously been successfully eradicated (Meng et al. 2015). In Europe, the two beetles were observed in more than 15 countries. Each country has developed its own eradication programme to reach the shared EU goals to eradicate both longhorned beetle species from the EU. Total costs are usually not directly available but the published numbers suggest that efforts were substantially lower than in North America (e.g. €3.35 million spent on five outbreaks in the period between 2001 and 2008 Haack et al. 2010). While several, mostly small outbreaks, were eradicated, in 2015 various populations of the two beetles are still under eradication in at least seven countries.^4^

## Public awareness and support

Public participation is an important but often overlooked component of effective invasive species management. Monitoring by the public may support discovery of IPPs, thereby facilitating early detection (e.g. Cacho et al. 2010). Public support for management strategies may also be needed to ensure smooth implementation, especially as often the management strategies are fairly destructive (Bertolino and Genovesi 2003; Simberloff et al. 2013). Awareness and support for managing IPPs could lead to changes in the public’s horticultural choices and enhancement of both legislation and management options, through consumer and political pressure (Stenlid et al. 2011). However, the threats may not be well understood due to problems in communicating some of the complex biological concepts related to pests and, especially, pathogens (e.g. hybridisation and mating types) as well as human role in IPP spread (Stenlid et al. 2011). These types of issues could be addressed by identifying gaps in public understanding and targeting communication accordingly. However, while public perceptions of invasive alien species have been examined (e.g. Bremner and Park 2007; Fischer and van der Wal 2007; Fischer et al. 2011; Sharp et al. 2011), there has been little research conducted on perceptions of IPPs in particular (Marzano et al. 2015).

Despite a potentially low awareness of details with regard to risks of IPPs, the public generally accepts the need to control invasive species, especially those perceived to be harmful (Bremner and Park 2007; Fischer and van der Wal 2007; Garcia-Llorente et al. 2008; Sharp et al. 2011). The public support of the control of invasive alien species depends on, for example, benefits/hazards associated with the species, but also to what extent the management method is humane (i.e. avoiding prolonged suffering), specific, safe and effective. The public, however, is less concerned with economic costs involved (Fraser 2006; Fitzgerald 2009). Although the public generally supports the control of invasive alien species, moderate measures are often supported more strongly than radical (even potentially more effective) measures (Sharp et al. 2011). People’s attitudes regarding species management are strongly influenced by their general value orientations (Bremner and Park 2007; Sharp et al. 2011), and although awareness or knowledge of non-native species have been found to be related to increase support for implementing management strategies (Bremner and Park 2007; Sharp et al. 2011), knowledge is generally a distal predictor of attitudes and behaviours (e.g. Ajzen 1991; see also Kaiser and Fuhrer 2003). Thus, higher awareness does not automatically lead to stronger support for effective management strategies or behavioural changes. Attitude theory (Ajzen 1991) furthermore makes a distinction between attitudes and behavioural intentions indicating that stronger support for management does not necessarily mean that the public will actively engage in issues related to IPPs or change their horticultural choices. Nevertheless, raising public awareness is highly important as a first step to involve the public. This is illustrated by the case study, summarised in Box 2, on efforts to counter ash dieback in the UK.

**Table:** 

**Box 2** Responses to Ash Dieback in the UK Ash dieback, which affects three ash species in Europe, i.e. European ash (*Fraxinus excelsior*), narrow-leaved ash (*F. angustifolia*) and *Fraxinus ornus* (Kirisits and Schwanda 2015) is caused by the fungus *Hymenoscyphus fraxineus* (anamorph *Chalara fraxinea*) (Baral et al. 2014), originating from Asia (Zhao et al. 2013). The disease was first discovered in Poland and Lithuania in the early 1990s, and has since been reported throughout much of northern and central Europe (Bakys et al. 2009; Gross et al. 2014). In March 2012, *H*. *fraxineus* was first reported in the UK in a nursery, in ash stock imported from The Netherlands. Infected plants were subsequently found in other nurseries and sites they supplied throughout England and Scotland. In late 2012, *H. fraxineus* was detected in the wider natural environment in south-eastern England. Surveys have since found the disease in woodlands and hedgerows as far north as north-east Scotland (www.forestry.gov.uk/infd-8w9euv). In rapid response to the discovery a multi-agency, cross-border Outbreak Management Team was formed and the Forestry Commission and other government staff were redeployed to undertake ash surveys across the UK. In October 2012, following a pest risk analysis conducted in consultation with the industry and affected parties, the UK Government passed emergency legislation to restrict ash imports and movement within Great Britain (www.forestry.gov.uk/infd-8yrdy7). A public awareness campaign was initiated to involve the public in searching for diseased ash, including widespread dissemination of information via channels such as the Forestry Commission website (www.forestry.gov.uk/chalara) and the media by researchers and officials. A smartphone application, Ashtag, was also quickly developed to harness public involvement for finding and mapping the disease’s distribution. Ashtag illustrates disease symptoms with a diagnostic guide and can be used for photographing and reporting new disease findings (www.ashtag.org). Hundreds of possible sightings of ash dieback have been reported through this system and checked by Forestry Commission officials. Public awareness of ash dieback in the UK and in other countries as a consequence of the media blitz is now high, perhaps partly due to the iconic nature of ash, which has increased public interest in its potential demise.	

The ash dieback case study shows that providing specific advice relevant to the public and general information on IPPs’ potential impacts on socially valued features of forests is likely to be successful. Public interest in ash dieback in the UK has also been used to highlight threats posed by invasive species to forestry in the UK, the importance of biosecurity and to strengthen calls for more care in labelling and importing live plants. This public awareness has bolstered government policy and led to establishment of the UK government’s Tree Health and Plant Biosecurity Task Force, which is reviewing biosecurity measures and considering further steps to prevent and manage future incursions. In conclusion, different combinations of multi-media public awareness campaigns using, for example, written information on the internet, apps, pamphlets and posters, but also in the form of computer games, PR products such as pens and mugs as well as TV and radio programmes are critical elements of strategies to manage forest pests and pathogens (Gardner and Stern 1996, for example the website by the University of Vermont)^5^.

## Discussion

Import of plants for planting and other high-risk commodities into the EU should be subject to stronger legislation to reduce the risk of introductions of invasive pests and pathogens. The separation between the regulation regarding plant health and biosecurity and invasive alien species does not increase the efficiency of prevention. However, the new regulation for alien invasive species and the revision of the regulation regarding plant health and biosecurity shows that the problem is gaining importance on the political agenda.

The pine wood nematode (PWN) example highlights the importance of early eradication attempts; strong enforcement of international agreements and legislation; and contingency plans (backed by the appropriate legislation and resources), which could have greatly enhanced the chances of successful eradication when PWN was first detected in Europe. However, efforts to control IPPs in the EU are currently constrained by a “Catch 22” dilemma since precautionary measures cannot be readily adopted without clear evidence of risk, which can only be obtained when damage has already occurred. There are also conflicts between Member States’ individual interests. Raising awareness of the risks, at all societal levels, will be critical to resolve these problems. Within EPPO and the NPPOs, there is an increasing shift from pest risk analysis towards pathway/commodity analyses. Since EPPO recommendations may be used by the EC, these changes may eventually be embedded in the legal framework. However, effective communication between science, policy makers and the general public will be essential to gain support for this shift and harmonise efforts of risk management and prevention.

Changes in policy and legislation might not directly be beneficial for some Member States, but would have major long-term benefits for the entire European Community. A major focus in these would be the need for collaboration within the EU (ideally coordinated by a central agency) to monitor, assess costs and benefits, contain and eradicate IPPs.

In an ideal situation, the EC should adopt harmonised precautionary measures, exploiting all available options to control IPPs. Recent infestations of the Pinewood Nematode, Asian and Citrus Longhorned Beetles and *Phytophthora* spp. have resulted in stronger scientific evidence to formulate pro-active legislation in contrast to the current reactive nature of the regulations in place. The national sovereignty of the Member States could restrict the range of possible actions to compromises acceptable to their constituents (national interests), which could be problematic as risks of invasions that have the potential to severely damage the forest industry are difficult to quantify. Thus, the EU may need to seek other protective strategies.

Current legislation regarding invasive species mostly outlines inspection schedules and methods, administrative protocols for responding to detected incursions, and potential containment and eradication steps. Thus, the regulatory framework promotes reactive, rather than precautionary measures. IPP control could be improved by shifting the regulatory focus from protection against specific invasive species towards securing commodities and potential invasion pathways; centralising responsibility for standardised implementation of EU legislation and raising public awareness. Some of these concerns have been addressed in the evaluation and subsequent revision of the legislation. However, the commitment to free trade and free movement of products and people within the EU will continue to inhibit the efficiency of the protections against IPPs.

### Shifting focus from species threat to commodities and potential invasion pathways

Prevention of invasions would need to concentrate on ‘safe’ commodities instead of marking certain commodities or origins as ‘un-safe’. This would mean that measures would address not only ‘expected’ pests and pathogens but also ‘unexpected’ or even ‘unknown’ invaders. Such a shift would require much greater legislative restrictions on imports of plants and plant products and, probably, transcontinental plant trade, as well as a stringent penalty system for violations (Mumford 2001). However, implementation of such ‘aggressive’ legislation would not be manageable under the current WTO system without changing the interpretation of risks under the framework. This would also provide stronger protection against new countries joining the WTO agreement, which should then be only allowed to import ‘safe’ commodities.

In addition to pathway analysis, a focus on commodities should be constituted by development of rigorous exploration and scientific documentation of the potential risks posed by commodities to enable acting on the precautionary principle. EPPO and the European NPPOs have to play a large role by compiling available data to provide valid and compelling arguments. A potential way to gather information and research the risks of potential IPPs on common commodities could be the use of sentinel nurseries (Roques et al. 2015); this method is also evaluated in the COST action that looks at a global network of these nurseries to functions as an early detection system. The idea is relatively simple by planting nurseries of common international species in several countries active in the live plant trade; the susceptibility to local pests should indicate the risk of local pest to the country of origin of the affected species. This could be a solution to the ‘Catch 22’ dilemma mentioned earlier in the text.

### A centralised response unit

A major constraint for actions to improve plant health in general is the Member States’ protection of national interests. As many serious IPPs were unknown or harmless in their native range before damage was detected in their introduced range, harmonisation of phytosanitary measures and regulatory legislation may be essential. Ideally, there should be optimal information exchange and collaboration between the organisational bodies regarding the EU phytosanitary system and management of invasive alien species (Unger 2005). The development of a central response unit has been suggested to reduce both the ecological threats (Hulme et al. 2009a) and conflicts between ecological and economic cost-benefits. Such a unit could harmonise efforts to prevent invasion of IPPs and invasive alien species in the EU, strengthen responses, and be responsible for long-term monitoring to prevent spread to other EU states. It could also include an emergency team to assess incursions and decide eradication and/or containment measures, financed by a general levy on plants or timber moved into or across the EU. Recently, Hantula et al. (2014) proposed a licencing system for plant trade, where market participants purchase a licence for a fee. The income of these licences could be used to fund monitoring and eradication costs and to reimburse the parties that have incurred economic damage as result of the measures against IPPs. Alternatively, costs of emergency measures could be borne by the importers responsible for incursions. For invasive species in general, it has been estimated that the costs of such an agency would be equivalent to <0.5 % of the annual cost of biological invasions in Europe (Hulme 2009).

### Raising and utilising public awareness

Involving the general public begins with information and education as deliberation of the public is the basis for democratic decisions (Carpini et al. 2004). Our example on ash dieback shows that attention in the media may trigger public engagement in a very specific case. However, the importance of awareness is the understanding of the wider concept behind the case in the public eye. In the recent years, citizen science has received increased attention, as it has been instrumental in collecting data over large spatial scales (Dickinson et al. 2012). Involvement of the public in detecting potential invasions of Asian Longhorned Beetle in the US included information campaigns targeting different groups with different materials (for example, the materials provided by the University of Vermont). However, the detection by citizens should not be used to replace monitoring by experts since misidentification might occur.

Public acceptance of implemented measures is critical, not least since the public will ultimately pay for them through increases in taxes or commodity prices (Hantula et al. 2014). Multi-media campaigns, with involvement of scientists and forest professionals, could help bring attention to recent introductions (species that may spread and effective means to control them). More systematic and strategically oriented communication, via, for example, email lists or newsletters to disseminate research findings to forestry professionals and policy makers, or seminars for forestry professionals and members of industry, would also be beneficial. It is important to identify the different levels stakeholders for successful utilisation of stakeholder support (Mumford 2002).

Where pest and pathogen impacts are clearly identifiable, citizen-science-based monitoring systems would be relevant to develop or explore in addition to any systematic monitoring at points of entry. This approach (used in efforts to combat ash dieback in the UK, Box 2; and detection of Asian Longhorned Beetle in the US) could enable cost-effective detection, enhance data collection and build support for management strategies. In addition, trade-marks for ‘safe’ and locally produced plants for planting and ornamentals (such as those used for wood- and fish-product certification systems) could be used to increase consumer awareness and increase pressure on industries to comply with associated standards (Marzano et al. 2015). By using a levy for trade or trade-licences (Hantula et al. 2014) for the most common vector of infestation, plants for planting, the costs of potential invasions will not just be carried by the actor that suffer the damage or consequences of eradication measures but shared within the whole sector.

## Conclusion

Public awareness is an important tool in the battle against IPPs that can be utilised in various ways. Even though 100 % protection against the risk of alien invasions is not realistic, still there is plenty of room for improvement in the different stages of introductions. Most importantly, the evaluation of the plant health regulation needs to be accompanied by a system that will provide funds for often costly measures for detection and eradication of invasions to retrieve some of the costs from the sector. This would relieve the economical burden of individual member states and could lead to a more readily response to invasions and potential to finance the central response unit needed to centralise the actions following invasions.

## Abbreviations

EC: European Commission
EPPO: European and Mediterranean Plant Protection Organisation
EU: European Union
GATT: General Agreement on Tariffs and Trade
IPPC: International Plant Protection Convention
IPPs: Invasive Pests and Pathogens
ISPM: International Standard for Phytosanitary Measures
NPPO: National Plant Protection Organisation
PWN: Pinewood Nematode
WTO: World Trade Organisation

## Footnotes

1 http://www.iufro.org/science/divisions/division-7/70000/publications/montesclaros-declaration/.

2 http://www.europe-aliens.org/.

3 http://www.fao.org/docrep/009/a0450e/a0450e00.htm all documents related to ISPM can be found here.

4 https://gd.eppo.int.

5 http://www.uvm.edu/albeetle/
